# The Psychophysiology of Action: A Multidisciplinary Endeavor for Integrating Action and Cognition

**DOI:** 10.3389/fpsyg.2018.01423

**Published:** 2018-08-29

**Authors:** Sven Hoffmann, Uirassu Borges, Laura Bröker, Sylvain Laborde, Roman Liepelt, Babett H. Lobinger, Jonna Löffler, Lisa Musculus, Markus Raab

**Affiliations:** ^1^Department of Performance Psychology, Institute of Psychology, German Sport University Cologne, Cologne, Germany; ^2^2EA 4260 Normandie Université, Caen, France; ^3^School of Applied Sciences, London Southbank University, London, United Kingdom

**Keywords:** psychophysiology, cognition, action, decision making, performance monitoring, embodiment

## Abstract

There is a vast amount of literature concerning the integration of action and cognition. Although this broad research area is of great interest for many disciplines like sports, psychology and cognitive neuroscience, only a few attempts tried to bring together different perspectives so far. Our goal is to provide a perspective to spark a debate across theoretical borders and integration of different disciplines via psychophysiology. In order to boost advances in this research field it is not only necessary to become aware of the different areas that are relevant but also to consider methodological aspects and challenges. We briefly describe the most relevant theoretical accounts to the question of how internal and external information processes or factors interact and, based on this, argue that research programs should consider the three dimensions: (a) dynamics of movements; (b) multivariate measures and; (c) dynamic statistical parameters. Only with an extended perspective on theoretical and methodological accounts, one would be able to integrate the dynamics of actions into theoretical advances.

## A Brief Definition of Action

Imagine Wladimir Klitschko, one of the most successful heavyweight boxing champions of all times, aiming at winning his next boxing match. To win the fight he has to plan and execute different actions that are embedded in a continuous stream of ongoing behavior. A precise definition of action is difficult given its manifold manifestations. Some actions can be characterized as ephemeral, like a hook during the fight. Other actions are more complex and enduring, like “winning the match,” and could be seen as a sum of sequential actions. The question of how to separate single actions is often solved by determining their underlying goals (Herwig et al., 2013; Künzell et al., 2017). In this vein, goals can either be within the perceptual range (e.g., hitting the opponent’s rib cage with the fist) or in anticipated future states that cannot yet be perceived in the environment (e.g., winning this match to become the leader of one’s weight division). According to Prinz (2013), goals can be “hot” and require active intervention to achieve them (e.g., sidestep to a counter strike) or they can be “cold” and temporally uncritical (e.g., increasing one’s stamina). Herwig et al. (2013) stated that “an action starts with the first behavioral activity directed toward a particular goal and ends with the achievement of the goal” (p. 106). To achieve a goal, external and internal factors must be geared toward the prospective goal state. For our Klitschko example, this would mean that actions might be influenced by internal factors like fitness level, accuracy, and speed of punches or by external factors such as the opponent being southpaw or noise and lighting conditions in the arena. In extension to the definition of Herwig et al. (2013), we propose that the dynamics of movements and behavioral activity, defined as one element of action besides goals, need to be further specified and partitioned. We suggest that a psychophysiological perspective on actions extends the existing definition by offering an explanation of how internal and external factors interact. Thus, a perspective on integrating action and cognition has to ask the questions: Which are the relevant mechanisms that initiate, guide, and evaluate action? And which methodological challenges arise when the aim is to integrate mind and motion? Answering these questions requires a consideration of different theoretical accounts, but also of new technical developments and methodological advances.

## Core Theoretical Accounts Related to the Interplay of Action and Cognition

### Multi-Sensory Integration

Most action is embedded in a rich perceptual environment and perceptual inputs coming from different input modalities have to be integrated to allow proper action selection. Multi-sensory integration is a crucial physiological theory that has, for instance, explained psycho-physiological phenomena such as the rubber hand illusion (Botvinick and Cohen, 1998) or the McGurk effect (McGurk and MacDonald, 1976). Multi-sensory integration addresses the perceptual binding problem (Milner, 1974; Mioche and Singer, 1989), which considers the question of how neural inputs from different modalities (e.g., vision, audition, olfaction) are integrated into one coherent, valid, and robust perceptual experience (Spence, 2011). The neurobiological bases of multi-sensory integration are bi- or multimodal neurons that are excited by more than one input modality existing in a large number of brain areas, such as the somatosensory cortex (Stein et al., 2009). Various complementing principles have been formulated for multi-sensory integration. The principle of visual dominance postulates that vision has a greater influence on the other senses than vice versa (Witten and Knudsen, 2005) the principle of modality appropriateness claims that cross-modal influences depend on the modality’s appropriateness for a given task (Welch and Warren, 1980). Finally, the principle of Bayesian integration postulates that the brain uses a form of Bayesian inference to integrate multimodal information into a coherent perception of the world (Deneve and Pouget, 2004).

### Embodied Cognition

The concept of embodied cognition assumes strong interactions between cognition, perception, and movement (Shapiro, 2010; Fischer and Coello, 2015), implying they cannot be studied independently of each other. In recent years, more and more evidence pointed to a considerable plasticity of the brain with respect to the integration of body parts (Blanke, 2012). Embodied cognition theories assume that the “self” emerges from the integration of bodily and/or environmental information. This information consists of visual, tactile, proprioceptive, vestibular, auditory, olfactory, visceral, and motor inputs (Blanke, 2012). Thus, embodied cognition is, at least from a neuroscience perspective, closely connected to multi-sensory integration. A recent study by Collins et al. (2016) showed that synchronizing stimulation of the hand somatosensory cortex and observed touches of a corresponding prosthetic hand created the perception of ownership of the artificial limb. From a psychological perspective, there is agreement that cognitive functions can only be understood when considering their relevance for actions (Wilson, 2002). To sum up, embodied cognition approaches postulate that cognitive processes are embodied and hypothesize that an individual’s bodily state (and its capacities and skills) and the environment interact (Rowlands, 2010).

### Decision Making

Prior to acting, one needs to decide to do so. However, theoretical concepts of decision making often neglect the fact that cognitive and motor processes are intertwined. Some approaches describe decisions as heuristics, with strategies formally defined as building blocks, i.e., a search-, stop-, and decision-rule. The mere composition of cognitive building blocks reveals that the involvement of the sensorimotor system is widely ignored. An exception to this is the simple heuristic approach, which claims that a simple heuristic applied to option generation and decision making in complex sports behavior considers the building block of execution (de Oliveira et al., 2014). The execution rule addresses the question of which action to carry out and, more importantly, how to execute it with the motor system (Raab et al., 2005; de Oliveira et al., 2014). In our opinion, the intertwined motor and cognitive components of decisions, which have been theoretically discussed and coined in the light of motor control (Wolpert and Landy, 2012) or “embodied choices” (Raab, 2017), should be empirically studied and incorporated into theories of the psychophysiology of movement. To reach this goal, psychophysiological models of motor heuristics and embodied choices have to be formulated. Different models of the psychophysiology of movement have been studied in animals (Cisek and Kalaska, 2010) and humans (Wichary and Smolen, 2016) and can serve as a starting point for theory testing, integration, and development. Now, neuroscientific studies integrating different methodological challenges (Kyathanahally et al., 2017) target decision making (Chand and Dhamala, 2017; Muraskin et al., 2017), and find correlations of decision making and somatosensory networks (Harris and Lim, 2016), however, the aspect of movement or “real” (i.e., dynamic) action is still rarely taken into account. We return to this point when outlining current methodological developments.

### Psychophysiology of Action and Cognitive Control

The literature concerning this topic is far too vast and complex to be laid down in detail here. Thus, we stick to the core mechanisms that seem relevant in this regard. From a neurophysiological point of view prefrontal cortex (PFC), motor cortex and basal ganglia networks and their corresponding neurotransmitter systems are crucial for action and cognition. More specifically, for task performance (“blocking and punching” to stick to the initial example) the stability of information in the PFC is crucial. Within these networks, there are structures and functions relevant for the evaluation of goal states. That is, for example, the allocation of attention to task-relevant features. On a rather simple level, the establishment of goals refers to efficient stimulus-response mappings concerning the task at hand, or, coming back to the initial example: “*Move right arm up if left shoulder of the opponent indicates a punch.*” But how do we keep track of our action goals on a neurophysiological level, and how are decisions implemented while acting? Working memory is crucial: the before-mentioned task-goal representations are being kept “online” within PFC networks (Jonides et al., 2008) via dopaminergic mediated activity states (Seamans and Yang, 2004). These assumptions match another idea: the reinforcement learning hypothesis (Holroyd and Coles, 2002). It assumes that within the PFC, dopamine (DA) is a kind of “gating signal” that is involved in keeping and adapting goal-state relations via reinforcement learning mechanisms (Miller, 2000). There exists considerable literature concerning connections of the PFC with parietal junctions (dorsolateral-prefrontal network) and how these networks control a variety of cognitive functions like motor planning, working memory, or allocation of attention. Ptak et al. (2017) have claimed that the core mechanism for motor planning (i.e., action planning) is action emulation. They argue that this emulation consists of a dynamic representation of abstract movement kinematics that sustains its internal manipulation. Thereby, it ensures its maintenance over short time periods. Further, it can be assumed that this dorsolateral prefrontal network has evolved from a motor control network to a general system supporting motor and cognitive functions. Related to the impact of actions, a recent account by Peterburs and Desmond (2016) suggests the cerebellum, the core structure for movement execution and motor adaptation, to play a crucial role in sensory prediction, error and conflict processing, response inhibition, as well as feedback learning. Here we return to the idea of embodied cognition: the key aspect of understanding cognition might be, not only from a psychological but also from a psychophysiological perspective, *action*. The neurophysiological mechanisms of action and the corresponding mechanisms related to the interaction with the environment are at the core of cognition.

### Interoceptive and Exteroceptive Changes

Finally, using our example with Klitschko, interoceptive and exteroceptive changes produced by actions can be considered dynamically interacting. For instance, muscle tension cannot be maintained after a series of punches (interoceptive change), and Klitschko’s opponent can positively influence the estimations of his chances to win by falling after a good punching sequence (exteroceptive change). One can argue that actions could be considered body movements which depend on external and internal factors. Such actions produce re-afferent feedback to interoceptive (generated within the body) and exteroceptive (generated outside the body) change. For instance, in a model of Schubotz (2007) these changes are meant to appear dynamically during movements. She defines interoceptive changes as proprioception (sense of the relative position of the body parts), visceroception (sense of the inner organs), equilibrioception (sense of balance) and nociception (sense from organs, joints, and bones). For complex actions, vision, audition, haptic and other senses are used to detect exteroceptive changes. Whereas we see a current trend in multi-sensory integration research (Greenlee, 2017), less knowledge is gained for the complex interaction of combining interoceptive and exteroceptive changes and their respective measures in one research program (Suzuki et al., 2013).

## Methodological Challenges Related to Integrating Action and Cognition

Besides the theoretical challenges that arise when taking the dynamics of actions into account, one of the core problems is the operationalization of the corresponding constructs. Nowadays there is a considerable amount of technical possibilities, however, only a few are used in dynamic contexts, or only in isolated paradigms measuring certain aspects. We think that more than static measurement of actions is required. For instance, in most classical experimental settings response times are measured or areas under the curve of a movement pattern are extracted, but temporal information provided by modern hard- and software is rarely taken into account. Further, these “static” measurements are often being analyzed independently of each other, e.g., response times are analyzed separate from EEG parameters, and are rarely integrated into attempts to solve theoretical questions (Debener, 2005; Hoffmann and Falkenstein, 2010; Plewan et al., 2016). Finally, many studies focus on central parameters like mean values or compare variances. However, in cognitive neuroscience a lot of measures have been developed which make it possible to describe the temporal dynamics of signals, e.g., time-frequency analysis, non-linear dynamics, or cross-coherence. We suggest a three-dimensional perspective integrating static and dynamic measurements, single and multiple measurements, and dynamics of statistics used. Anyway, the attempt to provide a comprehensive description of all possible methods would be too ambitious. In the following we will therefore describe essential aspects of this three-dimensional perspective and exemplify each aspect by selected methods.

### From Static to a Dynamic Measurement of Action

The use of “static” measurements has a long tradition in psychology and is closely connected to the concept of the measurement error (Lord, 1959; Novick, 1966; Rozeboom et al., 1969). In experimental research (as in any empirical research) two types of errors, systematic and random error, might arise. By controlling the experimental setup, one hopes to control for errors or at least keep them constant. This has been achieved with rather static devices: The measurement error (of the device) in simple button presses can be easily controlled. Also, measuring psychophysiological variables can be quite tricky due to the sensitivity of the systems to movement artifacts or other artifact sources. In psychophysiology the term “signal-to-noise ratio” describes this relation of true value and error. To keep this ratio at optimum, the influence of artifacts can be kept low if the participant does not move strongly. Related to the aspect of how to deal with artifacts and how to report them, precise recommendations exist (Keil et al., 2013). Anyway, in former days, measurement instruments did not have the same high precision modern systems have, e.g., eye-tracking systems with high. Also, it was not possible to measure “online,” that is, in more dynamic settings. This was due to the before mentioned problems, but also because it was not possible to measure remotely electromyographic or EEG data. In recent years solutions to these problems, for example, mobile EEG (Vos and Debener, 2014; Wascher et al., 2014) or even mobile brain/body imaging (Makeig, 2009; Banaei et al., 2017) have been put forward. Some of these systems show an excellent signal-to-noise ratio (Radüntz, 2018). However, many studies rather focus on “static” measurements like button presses to indicate choices. Only a few researchers integrate dynamic measures like eye-movements (Krajbich and Rangel, 2011). These dynamic measures could be highly relevant in order to model for example “true” action adaptation, like the change of direction of a movement during execution. We argue that due to this technical evolution more dynamic measurements should be taken into account since they provide a more fine-graded analysis of the mechanisms involved.

### From Single to Multiple Measurements

If one takes into account dynamic measurements, it seems straightforward to make corresponding inferences concerning the number of dependent variables that are being taken into account. Obviously, one has to be aware of the problem that: (a) one has to carefully select variables meaningful for the research question at hand and (b) to control for the problem of alpha error inflation. However, recent advances in statistics and neurosciences promise to integrate measures that might not only bring together different approaches but also provide new insights. One of these developments is, for example, the increasing usage of multivariate statistics to model artifact and neural sources (Jung et al., 2000). With these techniques it is even possible to integrate different measurements like simultaneous EEG-fRMI (Debener et al., 2006; Diukova et al., 2008; Sajda, 2009; Hoffmann et al., 2013; Dizaji and Soltanian-Zadeh, 2017). For a detailed and extended overview of methodological advances, the reader might refer to a recent research topic edited by Gramann et al. (2014).

### From Static to Dynamic Parameters

With “static” we refer to the assumption that the mean is an estimate of the “true” value of some operationalized concept. This assumption holds when any non-systematic variation is distributed normally around the “true” value and that with increasing sample size such variation approximates zero. Shortly described it is aimed to minimize the measurement error. For instance in response times experiments, the mean response times are assumed to estimate the “true response time” with respect to some experimental condition. The logic behind that is: If the cognitive (or neural) system responds to a stimulus, the response consists of the “true” response and the error. With increasing number of trials, this error is being statistically minimized. However, a vast amount of literature presenting alternative statistical parameters, capturing the variability of responses, has emerged. Indeed, this variability of responses might be modulated and might indicate variations in the processes of interest. One way to capture it are for example ex-Gaussians. They provide good fits of empirical response time distributions (Spieler et al., 1996; Matzke and Wagenmakers, 2009) and they allow descriptions of differences between conditions reflected in shifting and/or skewing of the RT distribution. Group differences can be more easily detected compared to classical approaches using Gaussian parameters like the mean. Other important approaches are drift-diffusion models (Ratcliff, 2013) and hierarchical drift-diffusion models (Lee and Wagenmakers, 2009; Wiecki et al., 2013). With drift-diffusion models one can basically model psychological parameters, e.g., information accumulation and decision threshold, by taking into account statistical parameters of response time distributions (Ratcliff, 2013). In sum, they have a long tradition in psychology, and recent advances integrate these accounts into neuroscience (Mulder et al., 2013; Forstmann et al., 2016).

## A Perspective on Integrating Cognition and Action

This headline promises much, and for sure a single, broad perspective will not provide a solution for “everything.” However, we believe that a research program related to the integration of action and cognition, be it related to embodiment or cognitive control, should at least consider the literature of the fields mentioned herein but also consider methodological and theoretical advances from other fields. Indeed, more and more studies integrate concepts and methods from different fields (Cohen and Cavanagh, 2011; Cavanagh et al., 2011; Schneider et al., 2014; Stock et al., 2016). For instance, Kiverstein and Miller (2015) described how an integration of neuroscience and embodiment could be laid down but there was a focus on the integration of emotion and cognition. Hence, the core ideas of embodiment, that the body *and* actions are relevant for cognition (Wilson, 2002), has not been derived in detail yet. Coming back to the initial boxing example, it gets clear that the question of how Klitschko got to some strategic decisions cannot be answered without considering the interaction of interoceptive and exteroceptive information. Also, the effect of cognition during the fight cannot be investigated without using dynamic and multivariate measures, since the interaction of these intero- and exteroceptive changes might depend exactly on that setting. Also, this situation may affect neural mechanisms of cognitive control. Having a look at the different streams of literature in the research areas of multi-sensory integration, embodied cognition, decision making, and the neural base of cognitive control, one can only conclude that the fields have to become aware of advances in the respective other fields. For example, there is a huge amount of research related to what decision making *is* and *why it works as it does* but the investigation of *how our neural system processes decisions* and *how all the processes are integrated “online”* has not yet been developed in depth. We think *that a psychophysiological perspective* on actions *extends existing definitions by offering the opportunity to find an explanation of how internal and external factors interact*. Any perspective on integrating action and cognition should strive to find answers to the question about the relevant mechanisms that *initiate, guide, and evaluate action.*

## Author Contributions

This perspective has been developed during a group retreat by the “Performance Psychology” group in 2017. Note that though the corresponding author coordinated, integrated, and wrote the manuscript, all other authors contributed equally with respect to conceptualization of the manuscript, as well as revising it. Thus, the order of the authors is, except the corresponding author, in alphabetical order.

## Conflict of Interest Statement

The authors declare that the research was conducted in the absence of any commercial or financial relationships that could be construed as a potential conflict of interest.
